# The temporal association of CapZ with early endosomes regulates endosomal trafficking and viral entry into host cells

**DOI:** 10.1186/s12915-024-01819-y

**Published:** 2024-01-25

**Authors:** Huazhang Zhu, Dawei Wang, Zuodong Ye, Lihong Huang, Wenjie Wei, Kui Ming Chan, Rongxin Zhang, Liang Zhang, Jianbo Yue

**Affiliations:** 1 City University of Hong Kong Shenzhen Research Institute, Shenzhen, China; 2 College of Veterinary Medicine, South China Agricultural University, Guangzhou, 510642 China; 3 Guangdong Laboratory for Lingnan Modern Agriculture, Guangzhou, 510642 China; 4 Research Core Facilities, Southern University of Science and Technology of China, Shenzhen, 518052 China; 5 Department of Biomedical Sciences, City University of Hong Kong, Hong Kong, China; 6 Laboratory of Immunology and Inflammation, Institute of Basic Medical Sciences and Department of Biotechnology, School of Life Sciences and Biopharmaceutics, Guangdong Pharmaceutical University, Guangzhou, China; 7 Divison of Natural and Applied Sciences, Synear Molecular Biology Lab, Duke Kunshan University, Kunshan, China

**Keywords:** CapZ, Endosomal trafficking, Early endosomes, Virus entry, Virus infection

## Abstract

**Background:**

Many viruses enter host cells by hijacking endosomal trafficking. CapZ, a canonical actin capping protein, participates in endosomal trafficking, yet its precise role in endocytosis and virus infection remains elusive.

**Results:**

Here, we showed that CapZ was transiently associated with early endosomes (EEs) and was subsequently released from the matured EEs after the fusion of two EEs, which was facilitated by PI(3)P to PI(3,5)P2 conversion. Vacuolin-1 (a triazine compound) stabilized CapZ at EEs and thus blocked the transition of EEs to late endosomes (LEs). Likewise, artificially tethering CapZ to EEs via a rapamycin-induced protein–protein interaction system blocked the early-to-late endosome transition. Remarkably, CapZ knockout or artificially tethering CapZ to EEs via rapamycin significantly inhibited flaviviruses, e.g., Zika virus (ZIKV) and dengue virus (DENV), or beta-coronavirus, e.g., murine hepatitis virus (MHV), infection by preventing the escape of RNA genome from endocytic vesicles.

**Conclusions:**

These results indicate that the temporal association of CapZ with EEs facilitates early-to-late endosome transition (physiologically) and the release of the viral genome from endocytic vesicles (pathologically).

**Supplementary Information:**

The online version contains supplementary material available at 10.1186/s12915-024-01819-y.

## Background

Endosomal trafficking, a vital cellular function, plays a role in a wide range of both physiological and pathological processes [1–3]. The endocytic system is a complex network of interconnected membrane-enclosed vesicles, including early endosomes, endosomal carrier vesicles/multivesicular bodies, recycling endosomes, and late endosomes. This system plays a crucial role in maintaining cellular balance and recycling energy. The process of endocytosis begins with the internalization of extracellular molecules, plasma membrane proteins, or membrane lipids. These internalized vesicles then merge with each other to form early endosomes. Early endosomes serve as sorting centers for the materials that have been internalized. Some of these materials are recycled back to the plasma membrane through recycling endosomes, while others progress into late endosomes. Eventually, late endosomes fuse with lysosomes for degradation. The regulation of endosomal trafficking is intricate and involves various components such as RAB small GTPases, tethering proteins, SNARE complexes, sorting nexins, and phosphoinositides, which are all part of interconnected signaling networks [4, 5]. How these signaling complexes are integrated to precisely regulate endosomal trafficking is poorly understood [5, 6].

In the signaling network regulating endosomal trafficking, various RAB proteins are recognized for their crucial contributions to defining endosome identity and progression. For example, the activation of RAB5 is indispensable for the maturation of early endosomes, while its deactivation is necessary for the transition from early to late endosomes. Nevertheless, the precise mechanisms responsible for the temporal and spatial regulation of RAB proteins remain unclear [7–10]. Likewise, the role of actin filaments in the internalization of endocytic vesicles has been documented, and a well-accepted knowledge is that the actin tails around the endocytic vesicles push these vesicles away from the plasma membrane [11–21]*.* Our recent findings actually argue against this dogma, that is, the massive actin filaments around endocytic vesicles not only hinder the movement of the endocytic vesicles after their internalization but also form a physical barrier to inhibiting homotypic fusion of these vesicles. CapZ (a stable CapZa-CapZb heterodimeric protein complex that caps the plus ends of actin filaments to stop filament assembly) helps to depolymerize these F-actin meshes around endocytic vesicles to facilitate fusion and movement. In addition, CapZ helps to recruit Rabaptin-5 (a RAB5 effector) and Rabex-5 (a RAB5 GEF) via its N-terminal domain to early endosomes when its C-terminal tail caps the F-actin filaments. In this way, it could activate RAB5, which, in turn, can attract more effectors or activators like Rabaptin-5 and Rabex-5. This creates a positive feedback loop that enhances RAB5’s activity even further [22, 23]. Yet, the precise role of CapZ, especially its temporal association with endosomes, in endosomal trafficking remains elusive.

Many animal viruses enter cells via receptor-mediated endocytosis mechanisms, including clathrin-mediated, micropinocytosis, caveolin/lipid raft-mediated, and phagocytosis. Once inside the endocytic vesicles, the viruses use endosomal cues, e.g., low pH, activation of proteases, and/or other unknown mechanisms, to fuse with the endocytic membrane, thereby transporting their genetic materials, either RNA or DNA, into the cytoplasm. The site and timing of the fusion/penetration of many viruses are well-regulated to deliver their genetic materials to ideal cytoplasmic locations for subsequent RNA or DNA replication. Different viruses might penetrate different endocytic vesicles, e.g., early endosomes, matured endosomes, late endosomes, or even endolysosomes. Therefore, the maturation of endocytic vesicles plays a vital role in the delivery of the viral genome into the cytoplasm [24–26].

Here, we investigated the role of CapZ in early-to-late endosome transition and the escape of the viral genome into the cytoplasm after internalization. We showed that the temporal association of CapZ with early endosomes is essential for the maturation of endocytic vesicles and viral infection of host cells.

## Results

### The release of CapZ from early endosomes coordinates early-to-late endosome transition

**Fig. 1:**
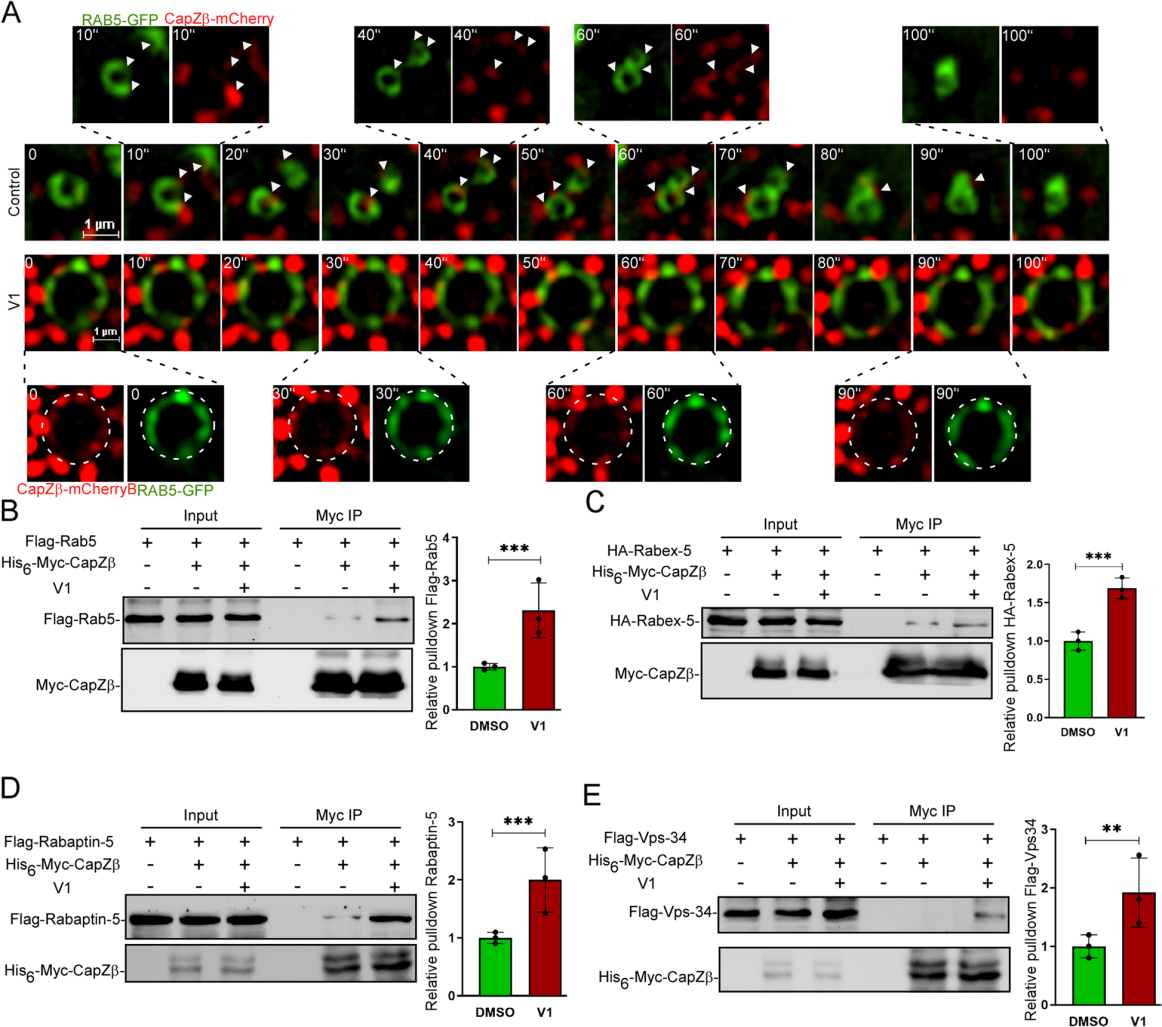
CapZ is transiently recruited to the early endosomes. **A** Live RAB5-GFP/CapZβ-mCherry-expressing HeLa cells treated with or without V1 (1 mM) were imaged using an N-SIM super-resolution microscope system (Movie S1 and S2), and selected frames of the time-lapse movies are presented here. The arrows in the upper panel indicate the transient association of CapZ puncta with RAB5-positive endosomes in control cells, and the dashed circle in the lower panel indicates the V1-induced tight association between CapZ and the enlarged RAB5-positive endosomes. Total 5 ~ 8 cells in either groups were imaged in 3 independent experiments. **B**–**E** Myc-CapZβ with Flag-Rab5 (**B**), HA-Rabex-5 (**C**), Flag-Rabaptin-5 (**D**), or Flag-Vps34 (**E**) were transiently transfected into HEK 293 T cells. The cell lysates were then incubated with anti-Myc-magnetic beads, and the pulldowns were subjected to immunoblot analysis against H.A.- or Flag-tag. The co-IP experiments were repeated three times independently. The difference between two groups was calculated using unpaired Student’s *t*-test. Differences were considered statistically significant when *P* < 0.05, *** *P* < 0.001

When early endosomes mature, RAB5 is gradually released and replaced by RAB7, thereby promoting the early-to-late endosome transition [10, 27]. We previously showed that CapZ facilitates the activation of Rab5 by recruiting Rabaptin-5 and Rabex-5 to early endosomes [22], and it is required for vacuolin-1 (V1, a triazine compound and potent inhibitor of endosomal trafficking)-induced RAB5 activation [23, 28, 29]. We, thus, characterized the temporal association of CapZ with endosomes in RAB5-GFP/CapZβ-mCherry-expressing HeLa cells treated with or without V1 by live-cell fluorescence imaging in an N-SIM S super-resolution microscope system. In control cells without V1 treatment, the association of CapZ to early endosomes was dynamic. First, CapZ was recruited to RAB5-positive early endosomes, then two early endosomes fused, and following this endosomal fusion, CapZ was released (top panel of Fig. 1A, and Additional file 1: Supplemental Video 1). However, in V1-treated cells, the association of CapZ with early endosomes was static as it appeared to be locked into enlarged RAB5-positive endosomes (bottom panel of Fig. 1A, and Additional file 2: Supplemental Video 2). Confocal imaging of CapZβ-mCherry-expressing HeLa cells treated with or without V1 confirmed that V1 significantly increased not only the colocalization of CapZ with RAB5 (Additional file 3: Fig. S1A), Rabaptin-5 (Additional file 3: Fig. S1A), and Rabex-5 (Additional file 3: Fig. S1B) but also the colocalization of RAB5 with Rabaptin-5 (Additional file 3: Fig. S1A) and Rabex-5 (Additional file 3: Fig. S1B). Coimmunoprecipitation (co-IP) experiments further confirmed that V1 markedly increased the interaction of CapZ with RAB5 (Fig. 1B), Rabex5 (Fig. 1C), Rabaptin5 (Fig. 1D), and Vps34 (Fig. 1E). These results suggest that the temporal association of CapZ with early endosomes participates in endosomal trafficking.

**Fig. 2:**
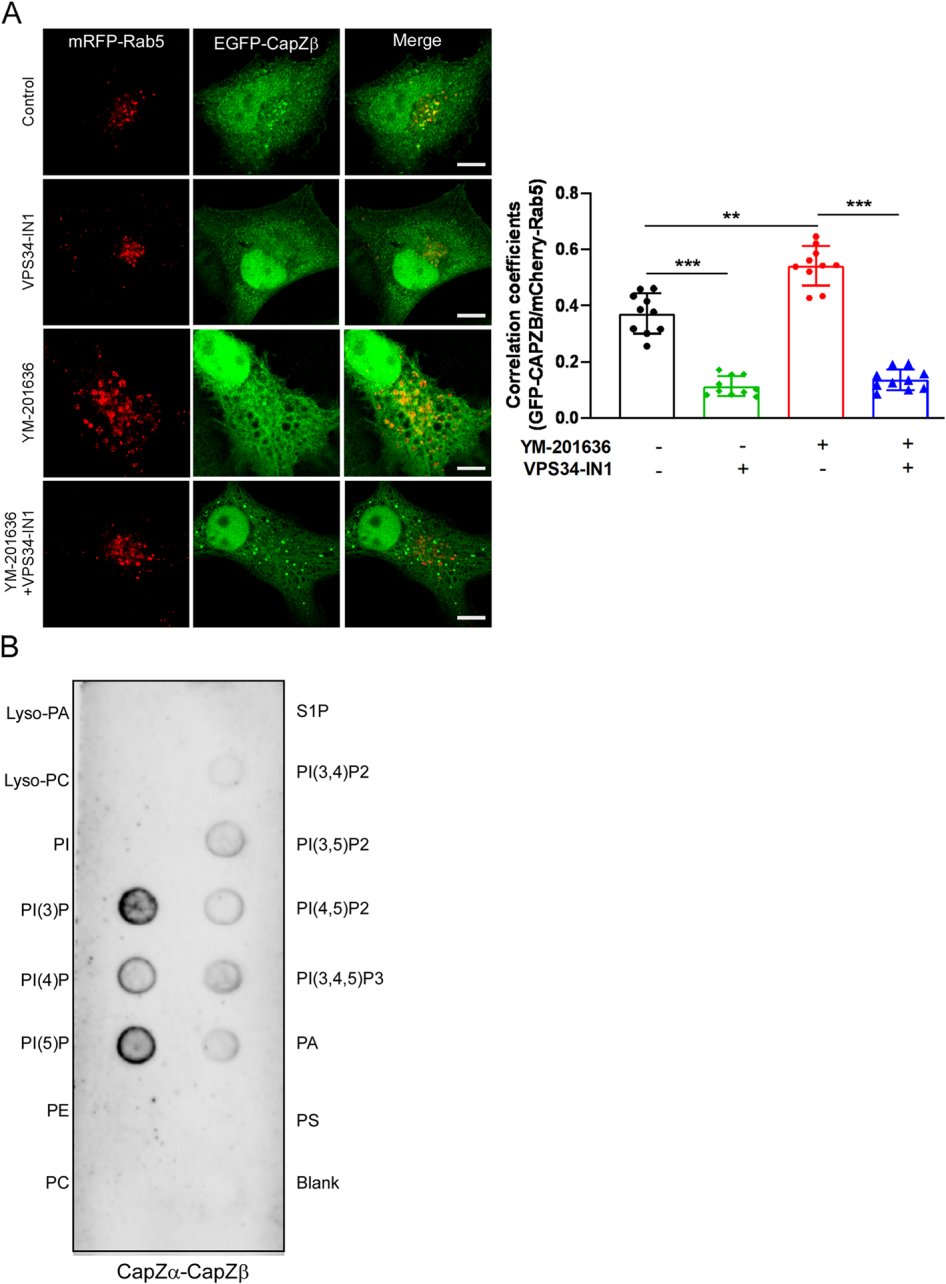
The CapZ complex interacts with phosphatidylinositol monophosphates. **A** CapZβ-GFP/RFP-RAB5A-expressing HeLa cells were pretreated with or without VPS34-IN1 (1 μM) for 1 h and were then treated with or without YM-201636 (5 μM) for 8 h, followed by confocal imaging. The scale bar is 5 μm. The colocalization coefficients (MCC) of mRFP-RAB5A/CapZβ-GFP were quantified. Data quantifications were analyzed using the ANOVA test and expressed as mean ± S.D. (*n* =  ~ 10 cells), *P < 0.05, **P < 0.01, ***P < 0.001. **B** The recombinant CapZ complex recognized phosphatidylinositol monophosphates (PI3P, PI4P, and PI5P) in a protein-lipid overlay (PLO) assay. The blots, images, and graphs represent data from at least three independent experiments

Interestingly, CapZ has been shown to bind with PI3P in vitro [30, 31]. The conversion of phosphatidylinositol 3-phosphate (PI(3)P) into phosphatidylinositol 3,5-bisphosphate (PI (3,5)P_2_), catalyzed by 5-phosphate kinase PIKfyve, is prerequisite for early-to-late endosome transition [32]. We showed that CapZ was closely associated with PI(3)P signals (Additional file 3: Fig. S2A). Therefore, we treated RFP-RAB5A/CapZβ-GFP-expressing HeLa cells with YM-201636 (a potent and selective PIKfyve inhibitor) [33] and found that YM-201636 not only enlarged early endosome but also markedly induced the colocalization between CapZ and RAB5. However, pretreatment of cells with VPS34-IN1 (a specific inhibitor of VPS34 which is responsible for PI3P generation) [34] abolished YM-201636-induced early endosome enlargement and the association of CapZ with RAB5 (Fig. 2A). We reasoned that the conversion of PI(3)P to PI (3,5)P_2_ might facilitate the release of CapZ from the matured early endosomes. To assess this possibility, we examined the binding of CapZ to phospholipids via a protein-lipid overlay (PLO) assay. The recombinant CapZa-CapZβ protein complex was co-purified from a large bacterial culture (Additional file 3: Fig. S2B).

We showed that the CapZ complex strongly interacted with phosphatidylinositol monophosphates (PI(3)P, PI(4)P, and PI(5)P), especially PI(3)P, but only weakly recognized PI (3,5)P_2_ (Fig. 2B). We speculate that the temporal association of CapZ with early endosomes might play a dichotomic role in controlling endosomal trafficking, with the initial association event being required for early endosome maturation [22, 23], whereas CapZ must be released for the subsequent early-to-late endosome transition.

### Stabilization of CapZ to early endosomes inhibits the early-to-late endosome transition

**Fig. 3:**
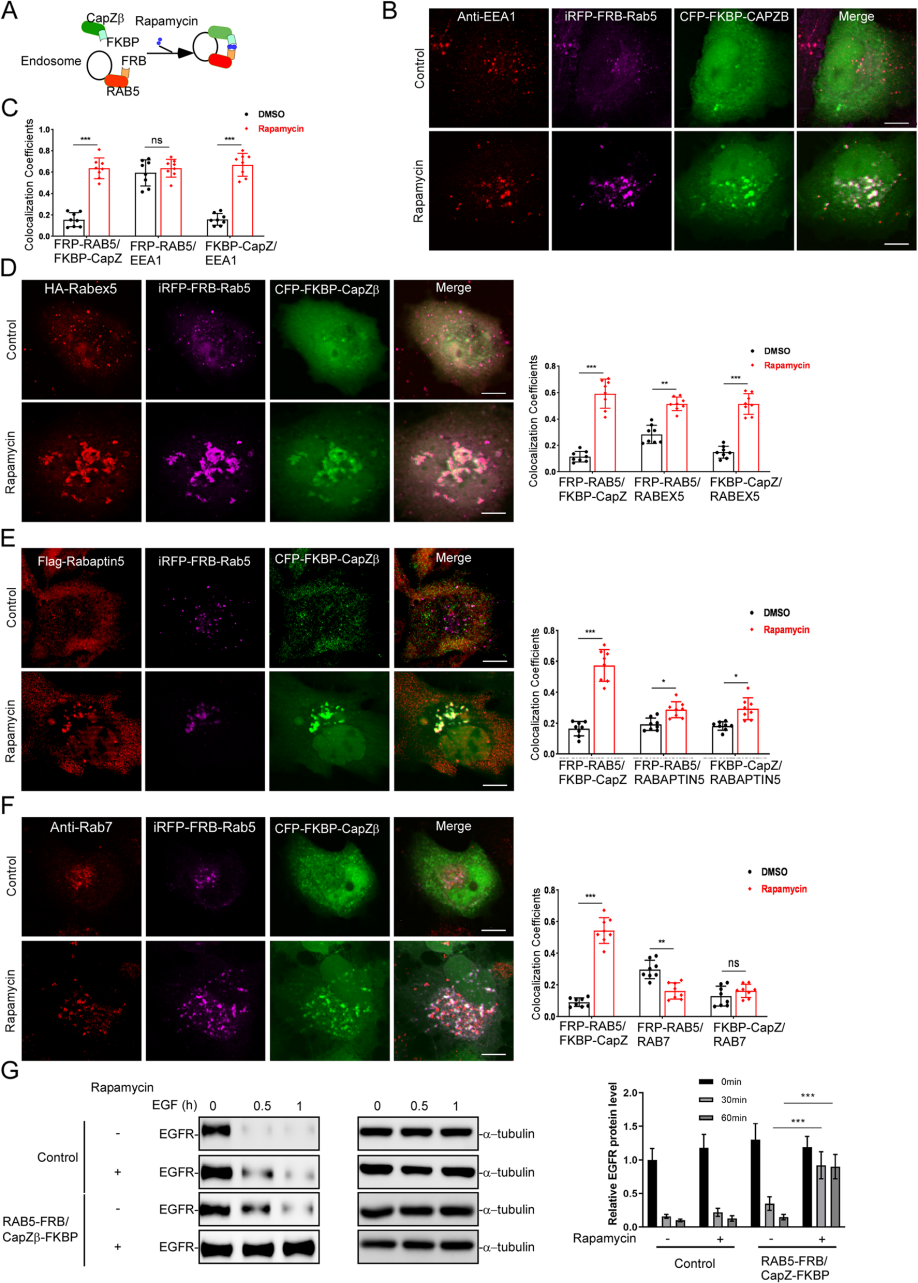
The artificial stabilization of CapZ on early endosomes inhibits endosomal maturation. **A** Schematic depiction of the strategy to induce the stable association of CapZ with early endosomes by conjugating RAB5 with FRB and CapZβ with FKBP in the presence of rapamycin (1 mM). **B**, **C** HeLa cells were transiently transfected with FRB-RAB5 and FKBP-CapZβ, and then they were incubated with rapamycin (1 mM) for 12 h to induce an interaction between RAB5 and CapZ, followed by anti-EEA1 immunostaining and confocal imaging (**B**). The colocalization coefficients (MCC) of RAB5A, CapZ, or EEA1 were quantified (**C**). **D**, **E** HeLa cells were transiently transfected with FRB-RAB5, FKBP-CapZβ, HA-Rabex-5 (**D**), and/or Flag-Rabaptin-5 (**E**); then, they were incubated with rapamycin (1 mM) for 12 h followed by anti-HA or anti-Flag immunostaining and confocal imaging. The colocalization coefficients (MCC) of RAB5A, CapZ, Rabex-5, or Rabaptin-5 were quantified. **F** HeLa cells were transiently transfected with FRB-RAB5 and FKBP-CapZβ, and then they were incubated with rapamycin (1 mM) for 12 h, followed by anti-Rab7 immunostaining and confocal imaging. The colocalization coefficients (MCC) of RAB5A, CapZ, or Rab7 were quantified. **G** Control or FRB-RAB5A/FKBP-CapZβ-expressing HeLa cells were incubated with or without rapamycin (1 mM) for 12 h, and they were then treated with EGF for 0 h, 0.5 h or 1 h. The cell lysates were subjected to EGFR immunoblot analysis. The scale bars are 5 μm. The blots, images, and graphs represent data from at least three independent experiments. Data quantifications were analyzed using unpaired Student’s *t*-test (two-group comparison in **C**, **D**, **E**, and **F**) or ANOVA (multiple comparisons in **G**) and expressed as mean ± S.D., *P < 0.05, **P < 0.01, ***P < 0.001

To study whether the release of CapZ from matured early endosomes is involved in the early-to-late endosome transition, we adopted a rapamycin-induced protein–protein interaction system by conjugating RAB5 with a rapamycin-binding motif of mTOR (FRB) and CapZβ with an FK506-binding protein (FKBP) (Fig. 3A) [35, 36]. We postulated that rapamycin-triggered tethering of CapZ to endosomes should, to a certain extent, recapitulate the V1 or YM-201636 treatment phenotype (Fig. 1, Fig. 2A, and Additional file 3: S1). Indeed, rapamycin treatment of the RAB5-FRB/CapZβ-FKBP-expressing cells resulted in enlarged RAB5-positive endosomes, and these endosomes exhibited strong colocalization with CapZ (Additional file 3: Fig. S3A). Quantifications of the confocal imaging of RAB5-FRB/CapZβ-FKBP-expressing cells treated with or without rapamycin further showed that rapamycin significantly induced not only the colocalization between RAB5-FRB and CapZβ-FKBP but also the association of CapZ with early endosomal proteins, e.g., EEA1 (Fig. 3B, C), Rabex5 (Fig. 3D), and Rabaptin5 (Fig. 3E). In contrast, rapamycin significantly decreased the colocalization of RAB5-FRB with late endosomal proteins, e.g., RAB7 (Fig. 3F) or LAMP1 (Additional file 3: Fig. S3B) in RAB5-FRB/CapZβ-FKBP-expressing cells. These results suggest that the artificial stabilization of CapZ with early endosomes inhibits the early-to-late endosome transition. Consistently, CapZ knockout resulted in the increase of lysosomal pH (Additional file 3: Fig. S3C).

The fusion between late endosome and lysosome is crucial for the degradation of internalized cargos such as EGFR [37]. We subsequently examined whether rapamycin treatment of the RAB5-FRB/CapZβ-FKBP-expressing cells affects EGFR degradation. In control cells, rapamycin treatment had very subtle effects on the degradation of the EGFR. However, in RAB5-FRP/CapZβ-FKBP-expressing cells, EGFR degradation was blocked by rapamycin treatment (Fig. 3G). These results confirm that the artificial stabilization of CapZ with early endosomes inhibits the early-to-late endosome transition.

The maturation of early endosomes is also essential for recycling endocytosis, such as the recycling of transferrin receptor (TfR) [38, 39]. We, thus, tested whether rapamycin treatment of the RAB5-FRB/CapZβ-FKBP-expressing cells affects TfR recycling by performing a fluorescent transferrin conjugates recycling assay. In this assay, after internalization of the iron-transferrin-TfR complex, iron is released from the endosomes into the cytoplasm. The transferrin-TfR complex is then recycled back to the plasma membrane, followed by the release of the fluorescent transferrin into the extracellular space. We showed that rapamycin had little effect on TfR recycling in control cells, but rapamycin treatment of RAB5-FRB/CapZβ-FKBP-expressing cells significantly inhibited the recycling of TfR when compared with cells without rapamycin treatment (Additional file 3: Fig. S4A, S4B). Since V1 markedly inhibited cell migration [23] and endosomal trafficking tightly regulated cell migration [40], we also performed the migration assay in control or RAB5-FRP/CapZβ-FKBP-expressing cells, treated with or without rapamycin. Rapamycin had little effect on migration in control cells, but significantly inhibited the migration of RAB5-FRP- and CapZβ-FKBP -expressing cells (Additional file 3: Fig. S4C). Collectively, these data indicate that the release of CapZ from matured early endosomes is required for the progression of endosomal trafficking.

### Disruption of early endosome maturation by CapZ deficiency inhibits flavivirus or mouse hepatitis virus (MHV) infection

**Fig. 4:**
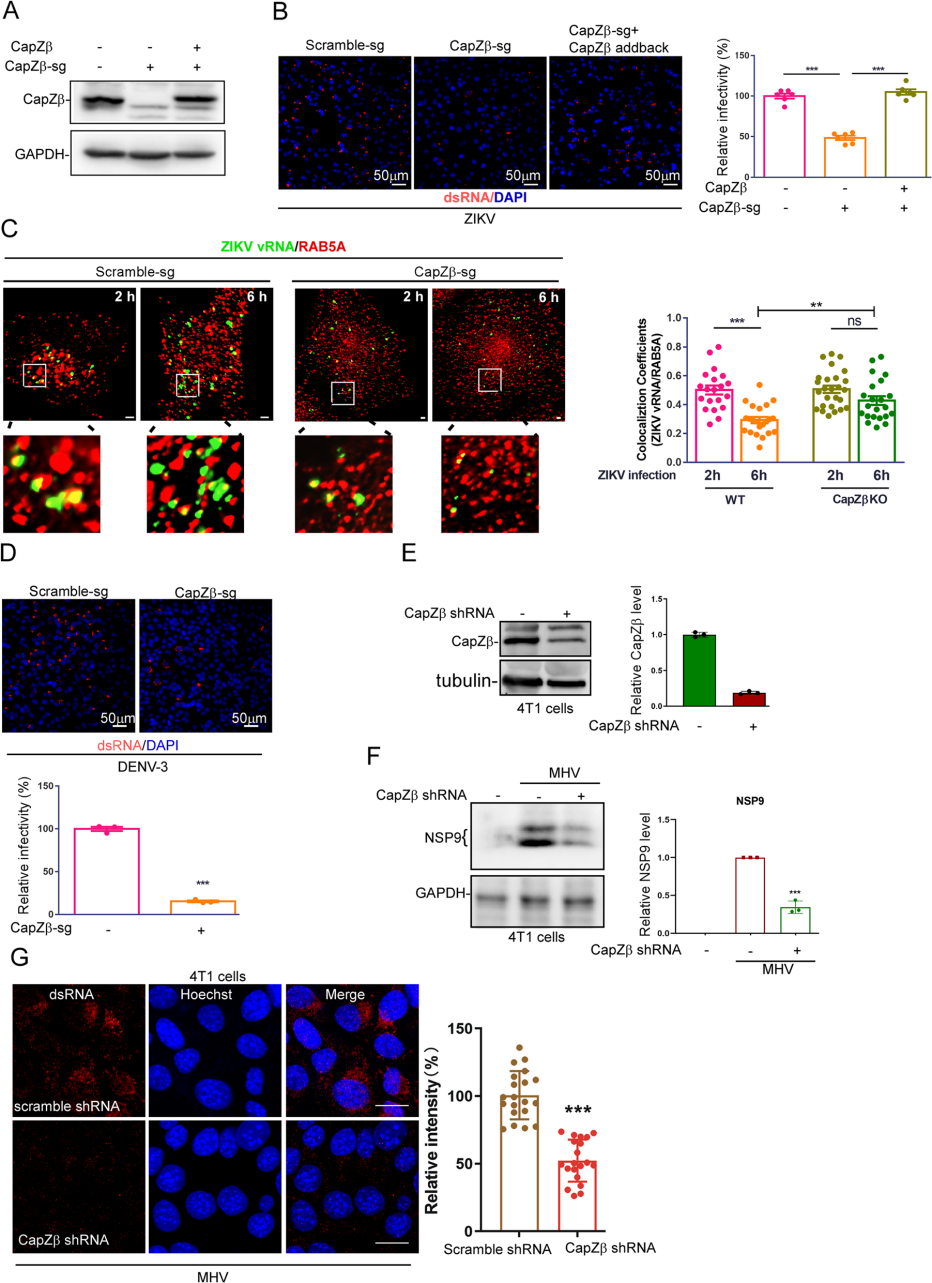
CapZ deficiency compromises the maturation of early endosomes to inhibit ZIKV, DENV, or MHV infection. **A** The expression of CapZβ in Control, CapZβ-knockout, or CapZβ-reconstituted HeLa cells. The CapZβ-reconstituted HeLa cells were constructed by stably transfecting pCDNA3.1-CapZβ-His_6_ into CapZβ-knockout cells. **B** Control, CapZβ-knockout, or CapZβ-reconstituted HeLa cells were infected with ~ 1 MOI ZIKV for 48 h. The cells were then immunolabeled with dsRNA antibodies and subjected to confocal imaging and analysis. The expression levels of CapZβ in these cells were also determined by immunoblot analysis. **C** Control or CapZβ-knockout cells were incubated with ~ 50 MOI ZIKV on ice for 1 h, and the cells were then incubated at 37 °C for indicated times, followed by in situ RNA hybridization of plus-strand RNA of ZIKV RNA genome and RAB5 immunostaining. The images were acquired by a confocal microscope and proceeded for colocalization analysis. The scale bar is 2 μm. **D** Control or CapZβ-knockout cells were infected with ~ 1 MOI DENV-3 for 48 h, and the cells were then immunolabeled with dsRNA antibodies and subjected to confocal imaging and analysis. **E** Lysates from Control or CapZβ-knockdown 4T1 cells were collected and subjected to SDS-PAGE followed by immunoblotting analysis against the indicated antibodies. **F**, **G** Control or CapZβ-knockdown 4T1 cells were infected with ~ 1 MOI of MHV for 24 h. The cells were either lysed and subjected to NSP9 and GAPDH immunoblot analysis (**F**) or were immunostained with dsRNA antibody followed by confocal imaging (**G**). The scale bar is 20 μm. The blots, images, and graphs represent data from at least three independent experiments. Data quantifications were analyzed using unpaired Student’s *t*-test (two-group comparison in **D**, **E**, **F**, and **G**) or ANOVA (multiple comparisons in **B**) and expressed as mean ± S.D., *P < 0.05, **P < 0.01, ***P < 0.001

Positive-sense single-stranded (+ ssRNA) viruses, e.g., flaviviruses (for example, Zika virus (ZIKV) and dengue virus (DENV)), or coronaviruses (for example, mouse hepatitis virus (MHV) and SARS-CoV-2), enter host cells via receptor-mediated endocytosis, and their genomes are then released from the early endosomes or/and late endosomes into the cytosol for subsequent viral genome replication [41, 42]. Therefore, we assessed the pathological relevance of CapZ-mediated endosomal trafficking in the context of flavivirus or MHV infection. First, we infected control, CapZβ-knockout, or CapZβ-reconstituted HeLa cells with ZIKV for 48 h and then performed the double-stranded RNAs (dsRNAs) immunostaining to quantify the ZIKV-infected cells. The dsRNAs are an intermediate in viral genome replication, produced by viral RNA polymerases following the infection of + ssRNA viruses, e.g., flaviviruses and coronaviruses [43–45]. We showed that ZIKV replication was significantly inhibited in CapZβ-knockout cells when compared to the control or CapZβ-reconstituted cells (Fig. 4A). To further examine which step(s) in ZIKV infection is/are affected by CapZ knockout, we incubated control or CapZβ-knockout HeLa cells with high titers ZIKV on the ice for 1 h, and then cultured the cells at 37 °C to initiate the internalization of the virus. At the indicated times after cold release, we fixed the cells and performed both in situ RNA hybridization to detect the positive-strand RNA of ZIKV (the viral genome (vRNA)) and RAB5 immunostaining to detect the early endosomes. As shown in Fig. 4B, 2 h after cold release, most ZIKVs were accumulated at early endosomes in both control and CapZβ-knockout cells, indicating that CapZ knockout does not affect the internalization of ZIKV. Similarly, we showed previously that CapZ knockout does not affect the internalization of EGFR [22]. Interestingly, 6 h after cold release, a significant amount of viruses already escaped from RAB5-positive endosomes in control cells; yet, in CapZβ-knockout cells, the majority of viruses were still sequestered in the early endosomes (Fig. 4B). Likewise, CapZβ knockout significantly inhibited DENV-3 infection in HeLa cells (Fig. 4C). We also knockdown the expression of CapZβ in 4T1 mouse breast cancer cells by shRNA (Fig. 4D) and showed CapZβ knockdown markedly inhibited MHV (a β-coronavirus) infection of 4T1 cells, manifested by the lower expression of nonstructural protein 9 (*NSP9*) (Fig. 4E) and lower dsRNA staining (Fig. 4F) in MHV-infected CapZβ-knockdown cells when compared to infected control cells. Nevertheless, these results indicate that CapZ deficiency compromises endosomes’ maturation, and this defect inhibits the release of + ssRNA viruses, e.g., ZIKV, DENV, or MHV, from endosomes, thereby rendering cells refractory to their infection.

### Artificial stabilization of CapZ to early endosomes inhibits ZIKV or MHV infection

**Fig. 5:**
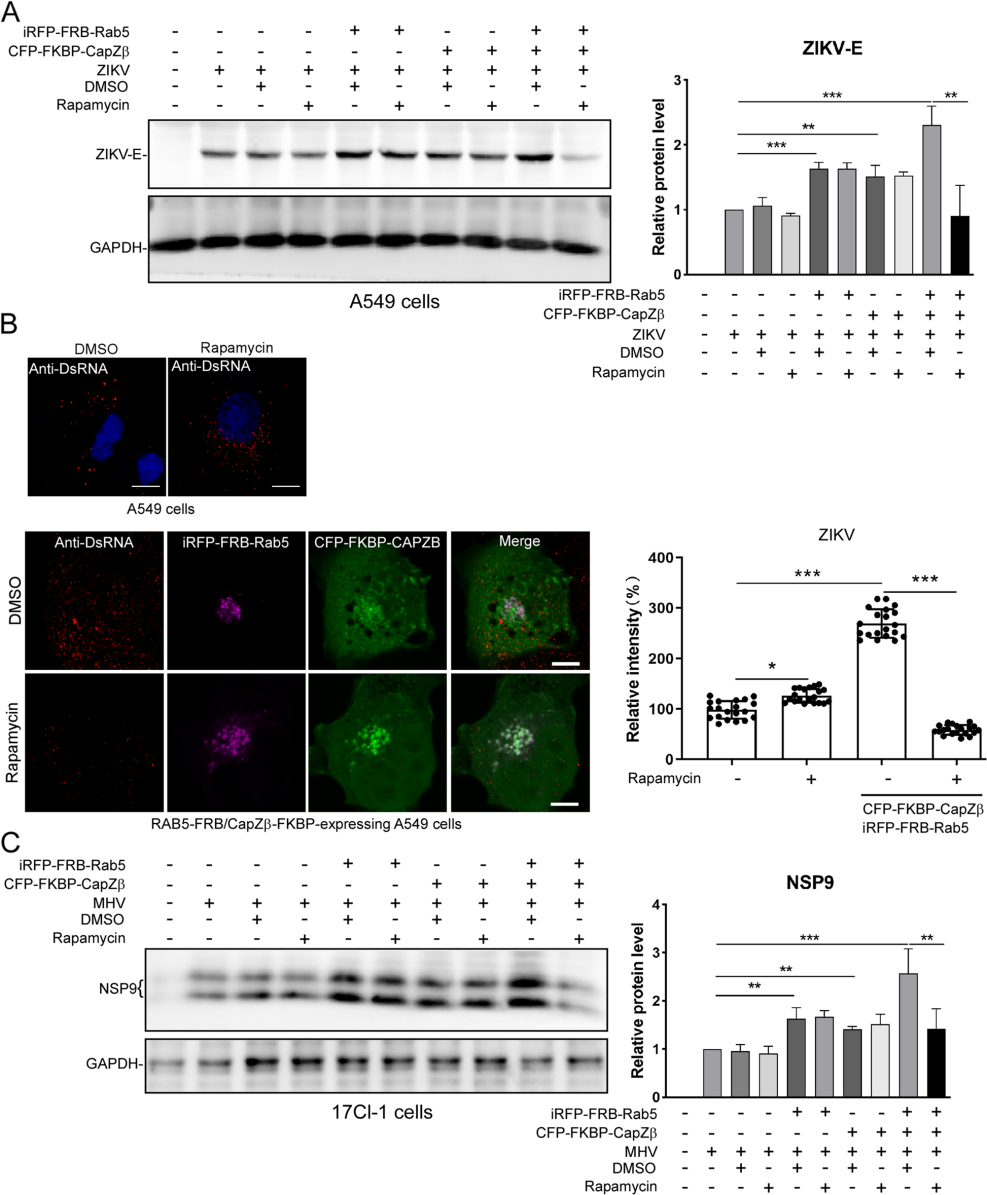
Stabilization of CapZ to early endosomes inhibits ZIKV or MHV infection. **A**, **B** A549 cells were transiently transfected with FRB-RAB5 and FKBP-CapZβ, then incubated with rapamycin for 12 h, followed by infection with ~ 1 MOI ZIKV for 48 h. The cells were lysed and subjected to ZIKV-E and GAPDH immunoblot analysis (**A**). Alternatively, cells were immunolabeled with dsRNA antibodies and subjected to confocal imaging and analysis (**B**). The scale bar is 5 μm. **C** 17CL cells were transiently transfected with FRB-RAB5 and FKBP-CapZβ, and they were then incubated with rapamycin for 12 h, followed by infection with ~ 1 MOI MHV for 48 h. The cells were lysed and subjected to Nsp9 and GAPDH immunoblot analysis. The blots, images, and graphs represent data from at least three independent experiments. The difference between the two groups was calculated using the ANOVA test. Differences were considered statistically significant when *P* < 0.05, ***P < 0.001

Since the release of CapZ is required for the early-to-late endosome maturation (Fig. 3, Additional file 3: S4 and Additional file 3: S3), we examined whether artificial stabilization of CapZ with early endosomes by rapamycin in RAB5-FRB/CapZβ-FKBP-expressing cells renders these cells refractory to ZIKV or MHV infection. We showed that rapamycin markedly inhibited the expression levels of ZIKV envelope (ZIKV-E) protein induced by ZIKV infection in RAB5-FRB/CapZβ-FKBP-expressing A549 cells, but not in control A549 cells (Fig. 5A). Likewise, rapamycin significantly inhibited dsRNA expression induced by ZIKV infection in RAB5-FRB/CapZβ-FKBP-expressing A549 cells, but not in control A549 cells (Fig. 5B). In addition, rapamycin markedly inhibited the expression levels of NSP9 induced by MHV infection in RAB5-FRB/CapZβ-FKBP-expressing 17Cl-1 cells, but not in control cells (Fig. 5C). In summary, these results indicate that the temporal association of CapZ with early endosomes participates in virus infection of host cells.

Besides V1, many compounds, e.g., chloroquine, bafilomycin, G42, and enanderinanin J, potently inhibit endolysosomal trafficking [46–50]. Therefore, we assessed the anti-ZIKV or MHV activity of some of these endolysosomal inhibitors. We showed that V1, chloroquine, and bafilomycin all markedly inhibited the expression levels of ZIKV-E protein induced by ZIKV infection (Additional file 3: Fig. S5A). These three compounds also significantly inhibited the virus production of ZIKV (Additional file 3: Fig. S5B). Likewise, these three compounds significantly inhibited NSP9 mRNA expression in MHV-infected 17Cl-1 cells (Additional file 3: Fig. S5C). Chloroquine or bafilomycin have been previously shown to inhibit ZIKV and/or MHV infection of host cells [47, 48]. Therefore, these results confirm that inhibition of endosomal trafficking effectively suppresses ZIKV or MHV infection.

## Discussion

Here, we showed that the association of CapZ with the RAB5-loaded early endosomes was transient as it was released from the matured earlier endosomes following the fusion of two endosomes (Fig. 1A and Additional file 1: Supplemental Movie 1). This was further supported by the observation that CapZ mainly recognized PI3P, the dominant phosphoinositides in early endosomes, not P.I. (3,5)P_2_, the main phosphoinositides in late endosomes (Fig. 2B, Additional file 3: S2A). Triazine compounds, such as V1, locked CapZ onto the endosomes and blocked the early-to-late endosome transition, manifested by the increase in RAB5 activity [28], the enlarged early endosomes (Fig. 1A, Additional file 3: S1), and increased interaction of CapZ with early endosomal proteins, e.g., RAB5, Rabex-5, Rabaptin-5, or Vps34 (Fig. 1B–E). Interestingly, our V1 treatment results are similar to those from previous reports, which demonstrate that the expression of a constitutively active (C.A.) RAB5 mutant enlarges endosomes by inducing the homotypic fusion of the early endosomes and blocking early-to-late endosome transition [10, 28]. Moreover, we showed that tethering CapZ to RAB5 with rapamycin in cells co-expressing CapZβ-FKBP and RAB5-FRB resulted in enlarged early endosomes, inhibited endocytosis, and decreased migration (Fig. 3A–G, Additional file 3: S3A, S3B, and Additional file 3: S4A-S4C). Therefore, these results suggest that the release of CapZ from the matured early endosomes is required for the early-to-late endosome transition.

As an enveloped virus, ZIKV, DENV, or MHV binds to its receptor(s) at the cell surface to start the receptor-mediated endocytosis. Thereafter, acidic pH and the activation of endosomal proteases, e.g., cathepsins, in the late endosomes facilitate the fusion of virus in endosomes, resulting in the release of viral genomic RNA into the cytoplasm of the host cells for subsequent viral RNA replication. Notably, lysosomotropic agents, e.g., chloroquine which can raise endolysosomal pH, or host protease inhibitors, e.g., cathepsin inhibitors, exhibit potent anti-viral activities (Additional file 3: Fig. S5) [51–54]. Here, we showed that CapZ knockout inhibited the release of ZIKV vRNA from endosomes, thereby inhibiting virus infection (Fig. 4A, B). The defects in endosome maturation caused by CapZ knockout might lead to the alkaline pH (Additional file 3: Fig. S3C) and inadequate processing of cathepsins in immature endosomes, thereby rendering these cells refractory to ZIKV infection. Likewise, we showed that tethering CapZ to early endosomes markedly inhibited ZIKV and MHV infections of host cells (Fig. 5). Therefore, our results suggest that inhibiting endosomal trafficking is an efficient therapeutic strategy against ZIKV, DENV, MHV, or other + ssRNA viruses. Along this line, Apilomod, an inhibitor of PIKfyve, potently inhibited flavivirus, Zaire ebolavirus, and SARS-CoV-2 infection [51, 52]. Here, we showed that V1, likely targeting CapZ and other endosomal proteins [23], potently inhibited ZIKV or MHV infections (Additional file 3: Fig. S5). Of note, V1’s cytotoxicity is lower than Apilomod [23, 55]. It is of interest to develop V1 analogs with improved specificity and efficacy against CapZ and apply them to treat virus infection.

## Conclusions

In summary, these results indicate that the temporal association of CapZ with EEs facilitates early-to-late endosome transition (physiologically) and the release of the viral genome from endocytic vesicles (pathologically).

## Methods

### Cell culture

HeLa, HEK 293 T, and A549 cells were ordered from ATCC. 4T1 cells were kindly provided by Dr. Minh LE. The following reagent was obtained through BEI Resources, NIAID, NIH as part of the Human Microbiome Project: 17Cl-1 cells. All the cells were cultured in DMEM (Invitrogen, 12,800–017) containing 10% fetal bovine serum (Invitrogen, 10,270–106) and 100 units/ml of penicillin/streptomycin (Invitrogen, 15,140–122) at 5% CO_2_ and 37 °C.

### Antibodies

The primary antibodies used in this study are listed as follows: GAPDH (Abclonal, AC002), CapZβ (Proteintech, 25,043–1-AP), His_6_-tag (ProteinTech, 66,005–1-Ig), α-tublin (Abclonal, AC012), RAB5A (CST, 3547), LAMP1 (CST, 9091), Rab7 (Abcam, ab137029), EEA1 (CST, 3288), Myc-tag (ProteinTech, 67,447–1-Ig), Flag-tag (Abclonal, AE005), HA-tag (Proteintech, 66,006–2-Ig), EGFR (Novusbio, AF231), NSP9 (GeneTex, GTX636839), Zikv-E (GeneTex, GTX133314), c-PARP (CST, 5625), DsRNA (Abcam, ab288755), goat anti-mouse IgG (H + L) secondary antibody (Invitrogen, 31,430), goat anti-rabbit IgG (H + L) secondary antibody (Invitrogen, 31,460).

### Plasmid construction

pRK5-mRFP-FYVE was a gift from Heike Fölsch (Northwestern University, USA), pRP-CapZβ-TwinStrep was a gift from Peter Barr-Gillespie (Addgene plasmid # 83,194), Flag-Vps34 was a gift from Qing Zhong ( addgene, #24,398), pSpCas9(BB)-2A-GFP was a gift from Feng Zhang(addgene,#48,138), iRFP-FRB-Rab5 was a gift from Tamas Balla (Addgene plasmid # 51,612), and EYFP-FKBP-PIP5K was a gift from Takanari Inoue (Johns Hopkins University); Flag-Rabaptin-5 was from MiaoLingBio,China (P8538).

pEGFP-N2-CapZβ-2xFKBP-EYFP or EYFP-FKBP-CapZβ was generated from the backbone of EYFP-FKBP-PIP5K by IGE BIOTECHNOLOGY, China (IG190608 or IG190453). CapZβ cDNA was amplified with PCR using FW primer (CCAAGCTTATGAGTGATCAGCAGCTGGA) and RV primer (GAGGTACCGATTATCAGGCTGGATGTAGAT) and then inserted between HindIII and KpnI restriction sites of the pENTR1A-GFP-N2 vector. After the LR recombination reaction, the target sequence flanked by attL sites is recombined into the lentiviral Gateway destination vector [56].

### CapZ knockdown or knockout

CapZβ sgRNA was cloned into pSpCas9(BB)-2A-GFP vector by using BbsI sites as previous described [57]. CapZβ shRNA was cloned into pLKO.1 TRC-cloning vector between AgeI and EcoRI sites followed the protocol provided by Addgene (https://www.addgene.org/protocols/plko/). The oligos sequences were attached below.

- CapZβ sgRNA-F CACCGCCAGGTCGATCAGGTCGCTG;
- CapZβ sgRNA-R AAACCAGCGACCTGATCGACCTGGC.
- CapZβ shRNA-F: CCGGGAACGAGATCTACTTTGGAAACTCGAGTTTCCAAAGTAGA TCTCGTTCTTTTTG;
- CapZβ shRNA-R: AATTCAAAAAGAACGAGATCTACTTTGGAAACTCGAGTTTCCAA AGTAGATCTCGTTC.

### Live cell image

Cells were plated on 35 mm glass-bottom dishes (Ibidi, 181,212/5) and transfected with RAB5-GFP and CapZβ-mCherry using lipofectamine 3000. After 24 h, the cells were treated with DMSO or V1 (1 μM) for 1 h. Images were obtained with an N-SIM super-resolution microscope system using a 100 × /1.40 NA oil objective lens. GFP and mCherry fluorescence were captured using 488 nm and 559 nm excitation filters, respectively. During imaging, the glass-bottom dishes were placed in a humidified chamber (Clamlide TC) supplemented with 5% CO_2_ at 37 °C. Images were captured using the “undelay” mode for 15 min, and after data acquisition, the raw images were processed with the Nikon NIS software.

### Immunofluorescence staining

Cells were fixed by 4% paraformaldehyde, followed by permeabilization with 0.1% Triton X-100 in PBS. After washing with PBS, the cells were incubated with 5% bovine serum albumin for 1 h at room temperature (R.T.) followed by incubation with the indicated primary antibodies at 4 °C overnight. The cells were washed again and incubated with the appropriate fluorescence-conjugated secondary antibody for 1 h at R.T. The cells were then mounted with ProLong™ Diamond Antifade mountant (Thermo Fisher, P36970). Images were acquired with the Zeiss LSM 880 confocal microscope and analyzed with the ZEISS ZEN microscopy software.

### In situ RNA hybridization

In situ RNA hybridization was performed by following the protocol of the RNAscope Multiplex Fluorescent Kit (Advanced Cell Diagnostics, 320,851) provided by the manufacturer. Briefly, cells on the coverslips were fixed with 4% PFA at R.T. for 15 min and then washed with PBS three times. After permeabilization with 0.3% Triton X-100 in PBS at R.T. for 1 h, the cells were incubated with a specific RNA probe targeted at ZIKV (ACD, 463,781) viral genome for 2 h at 40 °C, and four signal amplification systems were applied to detect target RNA. With a cascade of signal amplification, the viral RNA of ZIKV-infected cells was visualized with fluorescent dye. Following RNA hybridization, cells were subjected to immunofluorescence staining as described above.

### Immunoprecipitation (Co-IP)

HEK 293 T cells were transfected with the indicated plasmids. Twelve hours after transfection, the cells were treated with either DMSO or V1 (1 μM) for another 12 h. Cells were lysed on ice for 30 min in lysis buffer (50 mM Tris–HCl, pH 7.5, 150 mM NaCl, 1 mM EDTA, 1%Triton X-100, and protease inhibitor cocktail). After centrifugation at 13,000 rpm for 15 min at 4 °C, supernatants were collected and incubated with anti-c-Myc-magnetic beads (MedChemExpress, HY-K0206) at 4 °C overnight. The bound proteins were eluted with 2xSDS sample buffer and subjected to immunoblot analysis.

### Recombinant protein purification

His_6_-MBP-CapZα and His_6_-MBP-CapZβ were expressed separately in E.coli BL21(DE3) pLysS strain and induced by 0.5 mM IPTG. After centrifugation, cell pellets were resuspended in Buffer A (50 mM Tris–HCl pH 7.5, 500 mM NaCl, 0.05% β-mercaptoethanol, 1 mM EDTA, 1% Triton- × 100, and 1 mM phenylmethylsulphonyl fluoride) containing 1 U/mL DNase I (Thermo Fisher, 90,083) and lysed with a high-pressure cell crusher. The cellular debris was then removed by centrifugation for 1 h at 18,000 rpm. The resultant supernatants were mixed and applied to the HisPur™ Ni–NTA Superflow Agarose (Thermo Fisher, 25,214) column before washing with Buffer B (50 mM Tris–HCl pH 7.5, 500 mM NaCl, 0.05% β-mercaptoethanol and 20 mM imidazole). The flowthrough was applied to the same column twice, and His_6_-tagged proteins were eluted with Buffer C (50 mM Tris–HCl pH 7.5, 0.05% β-mercaptoethanol, 100 mM imidazole, and 500 mM NaCl). Eluted proteins were then applied to the Dextrin Sepharose™ ( Cytiva, 28–9355-97) column twice to remove the nonspecific proteins. The His_6_-MBP-CapZα and His_6_-MBP-CapZβ complexes were finally eluted with Buffer D (10 mM maltose in PBS pH 7.5). The eluted proteins were quantified by electrophoresis and Coomassie Brilliant Blue staining*.*

### Protein-lipid overlay assay

PIP Strips™ Membranes (Thermo Fisher, P23750) was blocked with 1% fatty acids-free BSA (Sigma-Aldrich, 126,575) for 1 h and incubated with the final concentration of 5 μg/mL recombinant His_6_-tagged CapZβ/CapZα in TBS with 0.1% Tween-20 (TBST) and 0.1% fatty acids-free BSA for 1 h at R.T. The membranes were then probed with anti-His_6_ (1:1.000; ProteinTech Group, 66,005–1-Ig) overnight at 4 °C, washed three times, and incubated with mouse HRP-linked IgG antibody (1:10,000; ProteinTech Group, SA00001-1) in TBST with 0.1% fatty acids-free for 1 h at R.T., followed by chemiluminescence detection and visualized by the Bio-Rad ChemiDoc MP Imaging System.

### Virus infection

A549 or 17Cl-1 cells were transfected with indicated plasmids using lipofectamine 3000 or electroporator, respectively. After 12 h transfection, cells were pretreated with either DMSO or rapamycin (1 μM) for 1 h, and infected with ~ 0.1 MOI of ZIKV for A549 cells or ~ 1 MOI MHV for 17Cl-1 cells. 4T1 cells were infected with ~ 1 MOI of MHV. Twenty-four hours post-infection, cells were fixed with 4% PFA, stained with an anti-dsRNA antibody, or lysed to harvest the total protein followed by immunoblot analysis.

### EGFR degradation assay

HeLa cells grown in 6-well plates were transfected with FRB-RAB5A and FKBP-CapZβ using lipofectamine 3000. Twenty-four hours post-transfection, cells were starved in the serum-free DMEM medium with DMSO or rapamycin (1 μM) overnight at 37 °C. The following day, EGFR endocytosis was initiated by adding EGF (100 μg /mL) (Thermo Fisher, PHG0311) in DMEM. Cells were harvested and lysed at the indicated time points and subjected to EGFR immunoblotting.

### Transferrin recycling assay

HeLa cells plated on glass coverslips in 24-well plates were transfected with FRB-RAB5A and FKBP-CapZβ using lipofectamine 3000. Twenty-four hours post-transfection, cells were maintained in complete medium overnight at 37 °C. After washing in ice-cold PBS, cells were incubated on ice in uptake medium (DMEM, 2% BSA, 20 mM HEPES, pH 7.5) containing transferrin-Alexa Fluor 594 (Thermo Fisher, T13343). One hour later, cells were washed in ice-cold PBS to remove the unbound ligand. Cells in one well were fixed to exhibit the total amount of bound ligands (time zero), and the cells in the remaining wells were replaced with warm complete medium and incubated at 5% CO_2_ and 37 °C for the indicated time, followed by fixation and confocal imaging.

### Quantitative RT-PCR (qPCR)

Total RNA was prepared using Trizol by following the manufacturer’s instructions. qPCR was performed with the One Step PrimeScript™ RT-PCR Kit (Takara, RR064B) in an Applied Biosystems™ ABI 7500 real-time PCR system. Target gene expression was quantified via the 2^−ΔΔCT^ method with 18S rRNA as the housekeeping gene. The results were presented as a relative value compared to the control group.

The primer sequences used are as follows:

**Table:** 

Target gene	Forward	Reverse
Mus-18S rRNA	CCGCGGTTCTATTTTGTTGGT	CTCTAGCGGCGCAATACGA
MHV N gene	CAGATCCTTGATGATGGCGTAGT	AGAGTGTCCTATCCCGACTTTCTC

### Statistical analysis

All statistical analyses were performed using Prism (GraphPad Software). The data were presented as mean ± S.D. The statistical significance of differences was determined by unpaired Student’s *t*-test (two groups comparison) or ANOVA (multiple comparisons). The asterisks indicate significance values *P* < 0.05 (*), *P* < 0.01 (**), and *P* < 0.001 (***); and *P* > 0.05 was considered to be not significantly different.

### Supplementary Information

**Additional file 1: Supplemental Video 1.** The N-Sim recording of Live RAB5-GFP/CapZβ-mCherry-expressing HeLa cells without V1 treatment.

**Additional file 2: Supplemental Video 2.** The N-Sim recording of Live RAB5-GFP/CapZβ-mCherry-expressing HeLa cells treated without V1 (1 mM).

**Additional file 3: Figure S1.** V1 induces the accumulation of CapZ on early endosomes. HeLa cells were transiently transfected with CapZβ-mCherry, RAB5A-BFP, and Rabaptin-5-GFP (A) or Rabex5-GFP (B), and treated with or without V1 (1 μM). The colocalization coefficients (MCCs) of CapZ/RAB5, CapZ/ Rabaptin-5, CapZ/Rabex-5, Rabex-5/RAB5, or Rabaptin-5/RAB5 were quantified. The images represent data from at least three independent experiments. The difference between two groups was calculated using an unpaired Student’s *t*-test. Differences were considered statistically significant when *P* < 0.05, *** *P* < 0.001. **Figure S2.** The role of CapZ in the early-to-late endosome transition. (A) CapZβ-GFP/RFP-FYVE-expressing (a PI(3)P sensor)/CapZβ-mCherry-expressing HeLa cells were subjected to confocal imaging, and the colocalization coefficients (MCCs) of mRFP-FYVE/CapZβ-EGFP were quantified. The scale bar is 5 μm. The images represent data from at least three independent experiments. (B) Coomassie staining of recombinant CapZa-CapZb complex purified from bacterial culture. The difference between the two groups was calculated using an unpaired Student’s *t*-test. Differences were considered statistically significant when *P* < 0.05, *** *P* < 0.001. **Figure S3.** CapZ participates in endosomal maturation. (A) HeLa cells were transiently transfected with FRB-RAB5 and FKBP-CapZβ, and then incubated with rapamycin (1 mM) for 12 h to induce an interaction between RAB5 and CapZ. (B) HeLa cells were transiently transfected with FRB-RAB5 and FKBP-CapZβ, and then they were incubated with rapamycin (1 mM) for 12 h, followed by anti-Lamp1 immunostaining and confocal imaging. (C) Control or CapZb knockout cells were labeled with lysosensor-Green DND-189, followed by confocal image and quantification. The scale bar is 5 μm. The colocalization coefficients (MCC) of RAB5A, CapZ, or Lamp1 were quantified. The difference between the two groups was calculated using an unpaired Student’s *t*-test. Differences were considered statistically significant when *P* < 0.05, *** *P* < 0.001. The images represent data from at least three independent experiments. **Figure S4.** The stabilization of CapZ on early endosomes inhibits endocytosis and cell migration. (A, B) Control or FRB-RAB5/FKBP-CapZβ expressing HeLa cells were incubated with or without rapamycin (1 mM) for 12 h, followed by incubation with transferin-594 on ice for 1.5 h. The cells were fixed at the time points indicated and processed for confocal imaging (A) and quantification (B). (C) Control or FRB-RAB5/FKBP-CapZβ expressing HeLa cells were placed into the upper chamber of a transwell plate in the absence or presence of rapamycin (1 mM). After 18 h, the cells in the lower chamber were stained with crystal violet and quantified. The difference between the two groups was calculated using the ANOVA test. Differences were considered statistically significant when *P* < 0.05, *** *P* < 0.001. The images and graphs represent data from at least three independent experiments. **Figure S5.** V1, chloroquine, and bafilomycin A1 inhibit ZIKV or MHV infection in host cells. (A, B) A549 cells were pretreated with V1, bafilomycin A1, and chloroquine for 3 h, and then infected with ~ 1 MOI of ZIKV for 24 h, followed by ZIKV-E and GAPDH immunoblot analysis (A) or the measurement of viral titers by TCID_50_ assay (B). (C) 17Cl-1 cells were pretreated with V1, bafilomycin A1 (50 nM), and chloroquine (25 mM) for 3 h, and then infected with ~ 1 MOI of MHV for 24 h, followed by the qRT-PCR analysis of MHV nsp9 mRNA expression. The blots, images, and graphs represent data from at least three independent experiments. The difference between the two groups was calculated using the ANOVA test. Differences were considered statistically significant when *P* < 0.05, *** *P* < 0.001. The blots and graphs represent data from at least three independent experiments

## Data Availability

All data are included in the main text and supporting data.

## Abbreviations

EE: Early endosome
LE: Late endosome
V1: Vacuolin-1
ZIKV: Zika virus
DENV: Dengue virus
MHV: Murine hepatitis virus
SNARE: Soluble *N*-ethylmaleimide-sensitive factor attachment protein receptor
