# Supplementary material for: The temporal association of CapZ with early endosomes regulates endosomal trafficking and viral entry into host cells
Source: BMC Biol. 2024 Jan 25;22:12. doi: 10.1186/s12915-024-01819-y (PMC10809671; doi:10.1186/s12915-024-01819-y)
Supplement: Supplementary file 3 — Additional file 3: Figure S1. V1 induces the accumulation of CapZ on early endosomes. HeLa cells were transiently transfected with CapZβ-mCherry, RAB5A-BFP, and Rabaptin-5-GFP (A) or Rabex5-GFP (B), and treated with or without V1 (1 μM). The colocalization coefficients (MCCs) of CapZ/RAB5, CapZ/ Rabaptin-5, CapZ/Rabex-5, Rabex-5/RAB5, or Rabaptin-5/RAB5 were quantified. The images represent data from at least three independent experiments. The difference between two groups was calculated using an unpaired Student’s t-test. Differences were considered statistically significant when P < 0.05, *** P < 0.001. Figure S2. The role of CapZ in the early-to-late endosome transition. (A) CapZβ-GFP/RFP-FYVE-expressing (a PI(3)P sensor)/CapZβ-mCherry-expressing HeLa cells were subjected to confocal imaging, and the colocalization coefficients (MCCs) of mRFP-FYVE/CapZβ-EGFP were quantified. The scale bar is 5 μm. The images represent data from at least three independent experiments. (B) Coomassie staining of recombinant CapZa-CapZb complex purified from bacterial culture. The difference between the two groups was calculated using an unpaired Student’s t-test. Differences were considered statistically significant when P < 0.05, *** P < 0.001. Figure S3. CapZ participates in endosomal maturation. (A) HeLa cells were transiently transfected with FRB-RAB5 and FKBP-CapZβ, and then incubated with rapamycin (1 mM) for 12 h to induce an interaction between RAB5 and CapZ. (B) HeLa cells were transiently transfected with FRB-RAB5 and FKBP-CapZβ, and then they were incubated with rapamycin (1 mM) for 12 h, followed by anti-Lamp1 immunostaining and confocal imaging. (C) Control or CapZb knockout cells were labeled with lysosensor-Green DND-189, followed by confocal image and quantification. The scale bar is 5 μm. The colocalization coefficients (MCC) of RAB5A, CapZ, or Lamp1 were quantified. The difference between the two groups was calculated using an unpaired Student’s t-test. Differences [file 12915_2024_1819_MOESM3_ESM.pdf]

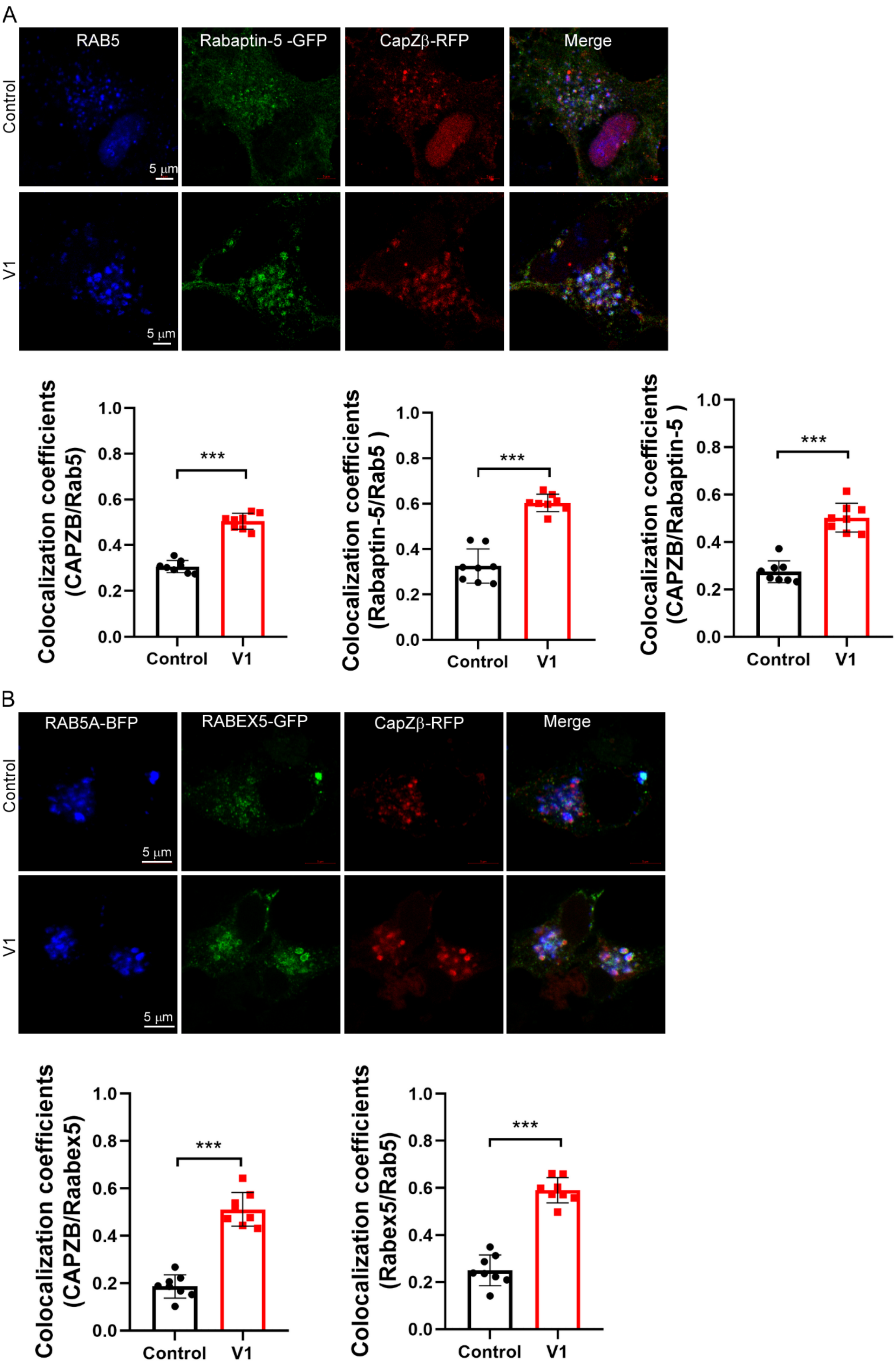
A
RAB5
Rabaptin-5 -GFP
CapZβ-RFP
Merge
Control
V1
5 μm
5 μm
Colocalization coefficients (CAPZB/Rab5)
Colocalization coefficients (Rabaptin-5/Rab5)
Colocalization coefficients (CAPZB/Rabaptin-5)
1.0
0.8
0.6
0.4
0.2
0.0
***
Control
V1
B
RAB5A-BFP
RABEX5-GFP
CapZβ-RFP
Merge
Control
V1
5 μm
5 μm
Colocalization coefficients (CAPZB/Raabex5)
Colocalization coefficients (Rabex5/Rab5)
1.0
0.8
0.6
0.4
0.2
0.0
***
Control
V1

Figure S1. V1 induces the accumulation of CapZ on early endosomes. HeLa cells were transiently transfected with CapZβ-mCherry, RAB5A-BFP, and Rabaptin-5-GFP (**A**) or Rabex5-GFP (**B**), and treated with or without V1 (1μM). The colocalization coefficients (MCCs) of CapZ/RAB5, CapZ/ Rabaptin-5, CapZ/Rabex-5, Rabex-5/RAB5, or Rabaptin-5/RAB5 were quantified. The images represent data from at least three independent experiments. The difference between two groups was calculated using an unpaired Student's t-test. Differences were considered statistically significant when $P<0.05$, *** $P<0.001$.

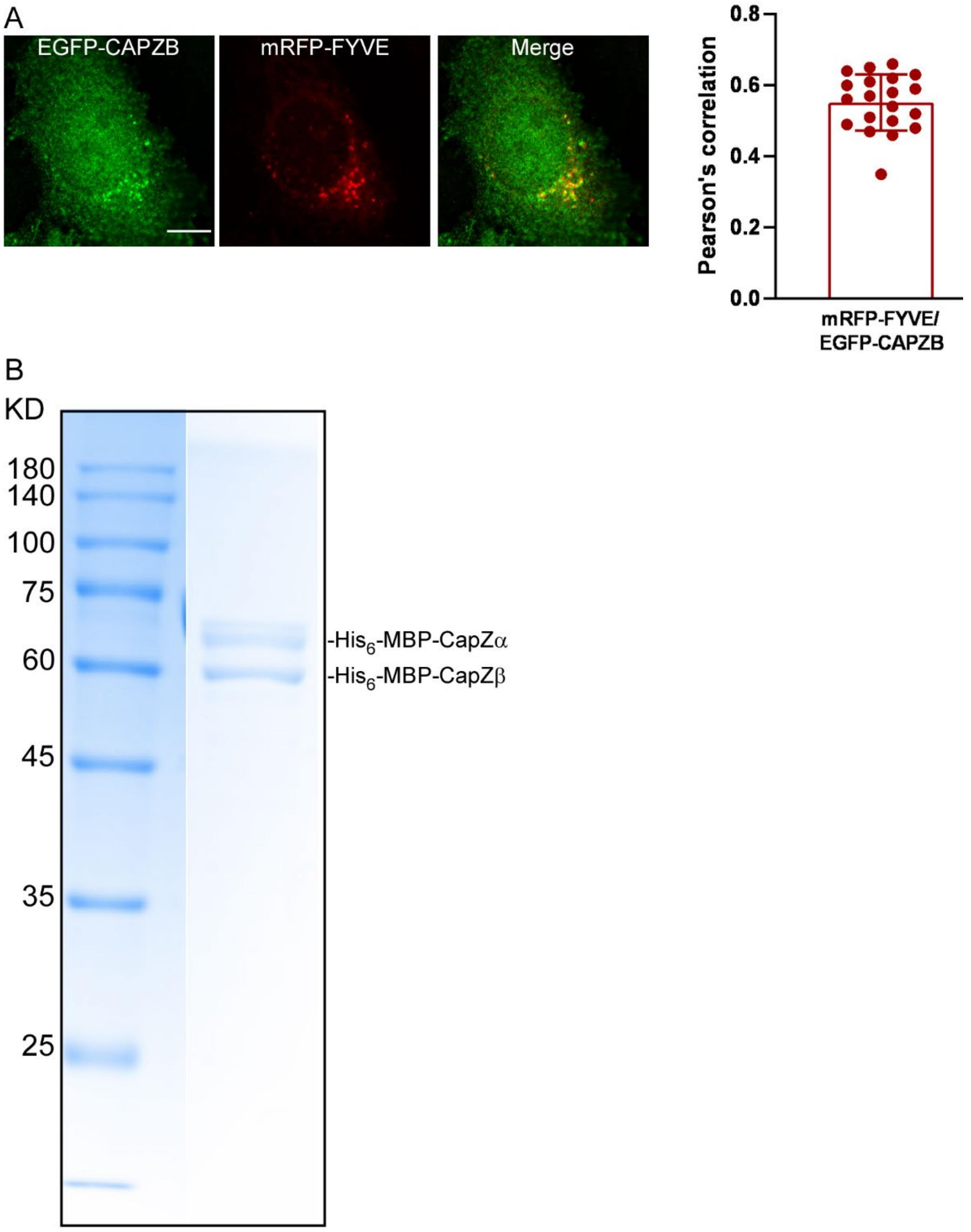

Figure S2. The role of CapZ in the early-to-late endosome transition. (A) CapZβ-GFP/RFP-FYVE-expressing (a PI(3)P sensor)/CapZβ-mCherry-expressing HeLa cells were subjected to confocal imaging, and the colocalization coefficients (MCCs) of mRFP-FYVE/CapZβ-EGFP were quantified. The scale bar is 5 µm. The images represent data from at least three independent experiments. (**B**) Coomassie staining of recombinant CapZα-CapZβ complex purified from bacterial culture. The difference between the two groups was calculated using an unpaired Student's t-test. Differences were considered statistically significant when $P<0.05$, *** $P<0.001$.

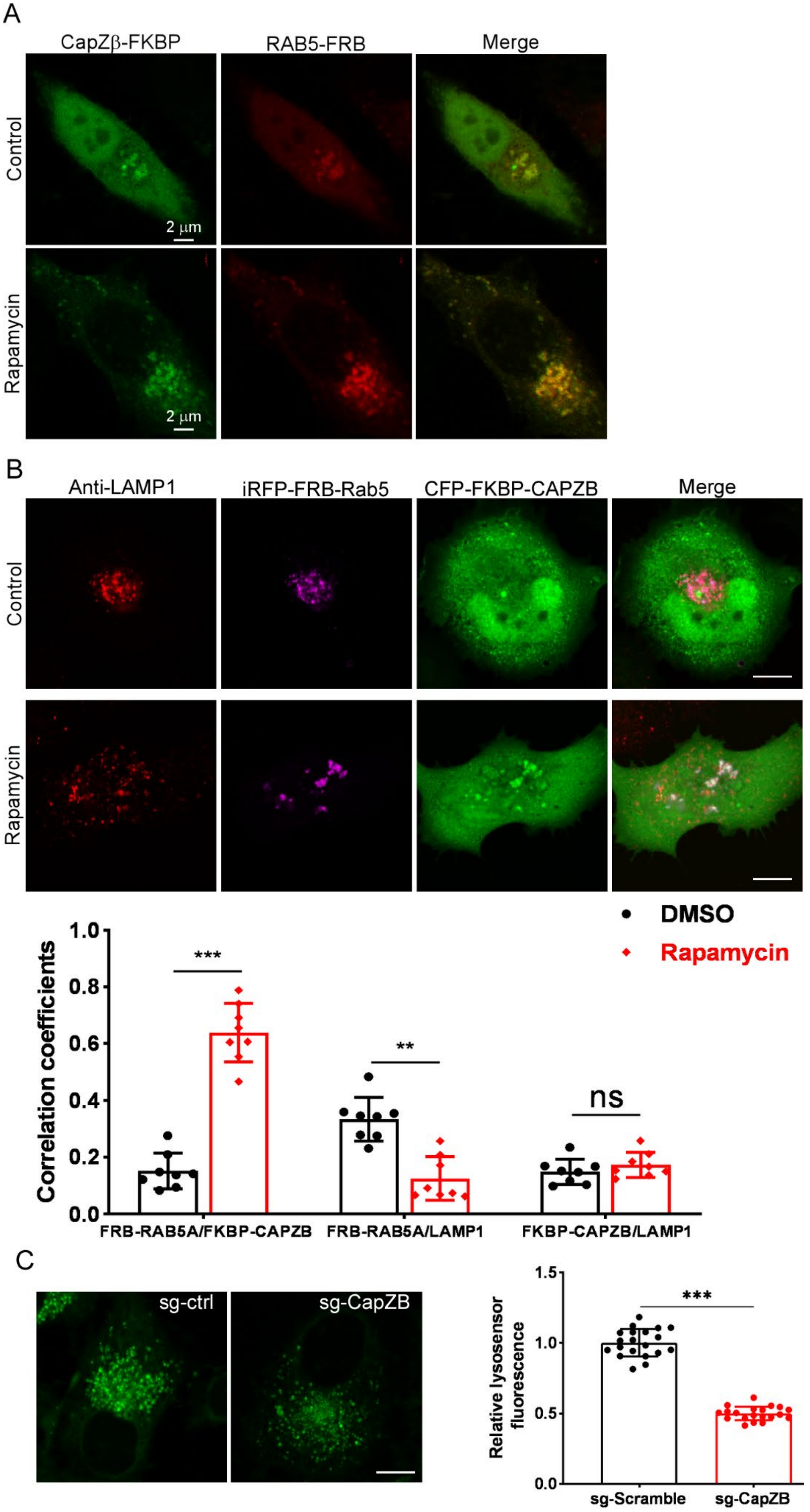
A
CapZβ-FKBP
RAB5-FRB
Merge
Control
Rapamycin
2 μm
2 μm
B
Anti-LAMP1
iRFP-FRB-Rab5
CFP-FKBP-CAPZB
Merge
Control
Rapamycin
DMSO
Rapamycin
Correlation coefficients
***
**
ns
FRB-RAB5A/FKBP-CAPZB
FRB-RAB5A/LAMP1
FKBP-CAPZB/LAMP1
C
sg-ctrl
sg-CapZB
Relative lysosensor fluorescence
***
sg-Scramble
sg-CapZB

Figure S3. CapZ participates in endosomal maturation. (A) HeLa cells were transiently transfected with FRB-RAB5 and FKBP-CapZβ, and then incubated with rapamycin (1 μM) for 12 h to induce an interaction between RAB5 and CapZ. (**B**) HeLa cells were transiently transfected with FRB-RAB5 and FKBP-CapZβ, and then they were incubated with rapamycin (1 μM) for 12 h, followed by anti-Lamp1 immunostaining and confocal imaging. **(C)** Control or CapZb knockout cells were labeled with lysosensor-Green DND-189, followed by confocal image and quantification. The scale bar is 5μm. The colocalization coefficients (MCC) of RAB5A, CapZ, or Lamp1 were quantified. The difference between the two groups was calculated using an unpaired Student's t-test. Differences were considered statistically significant when $P<0.05$, *** $P<0.001$. The images represent data from at least three independent experiments.

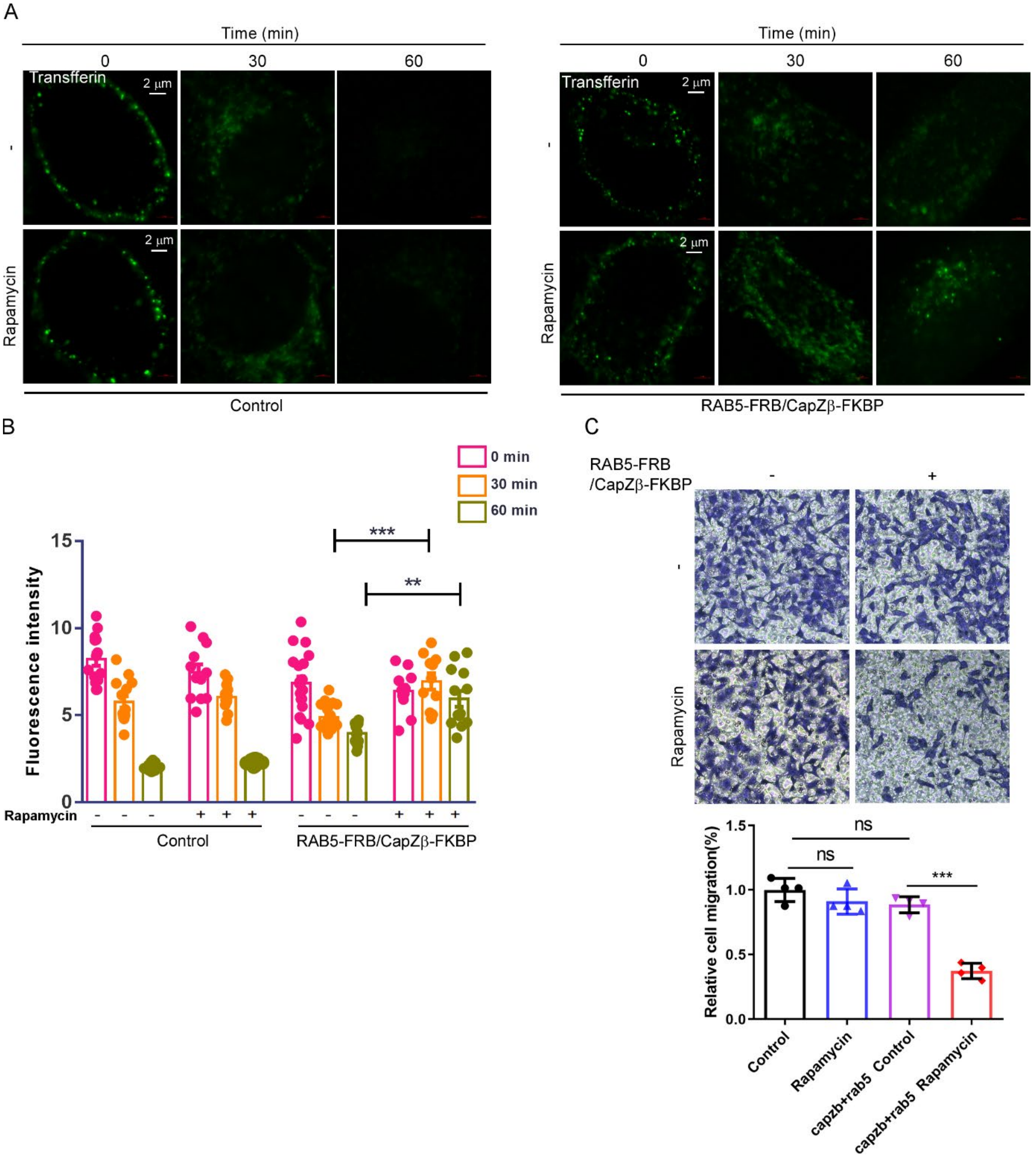

Figure S4. The stabilization of CapZ on early endosomes inhibits endocytosis and cell migration. (**A, B**) Control or FRB-RAB5/FKBP-CapZβ expressing HeLa cells were incubated with or without rapamycin (1 μM) for 12 h, followed by incubation with transferin-594 on ice for 1.5 h. The cells were fixed at the time points indicated and processed for confocal imaging (**A**) and quantification (**B**). **(C)** Control or FRB-RAB5/FKBP-CapZβ expressing HeLa cells were placed into the upper chamber of a transwell plate in the absence or presence of rapamycin (1 μM). After 18 h, the cells in the lower chamber were stained with crystal violet and quantified. The difference between the two groups was calculated using the ANOVA test. Differences were considered

statistically significant when P<0.05, *** P<0.001. The images and graphs represent data from at least three independent experiments.

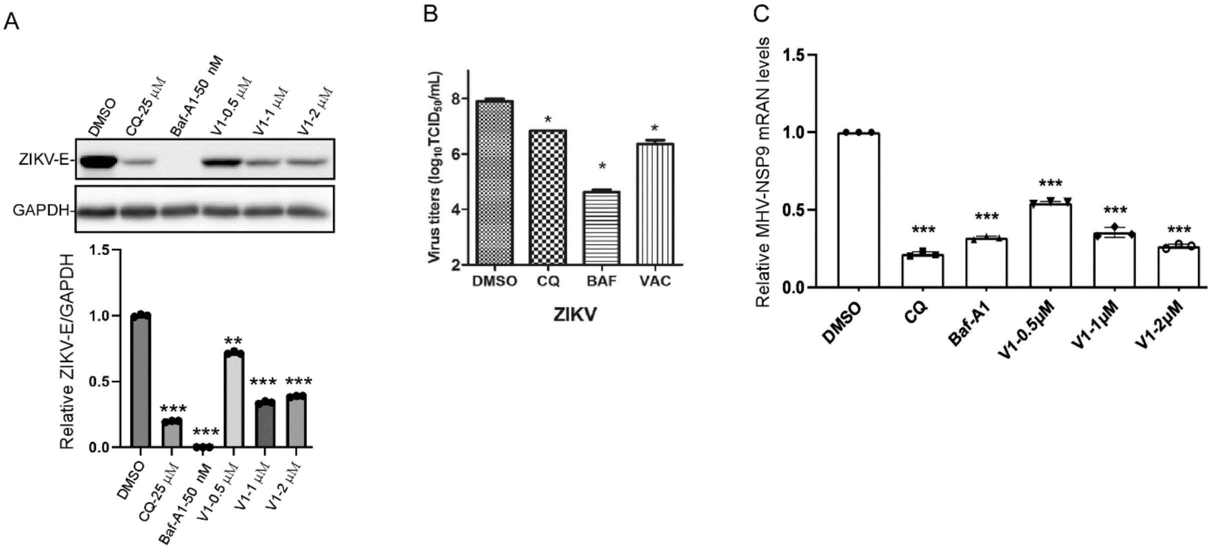

Figure S5. V1, chloroquine, and bafilomycin A1 inhibit ZIKV or MHV infection in host cells. (**A, B**) A549 cells were pretreated with V1, bafilomycin A1, and chloroquine for 3 h, and then infected with ~1 MOI of ZIKV for 24 h, followed by ZIKV-E and GAPDH immunoblot analysis (**A**) or the measurement of viral titers by $TCID_{50}$ assay (**B**). (**C**) 17Cl-1 cells were pretreated with V1, bafilomycin A1 (50 nM), and chloroquine (25 μM) for 3 h, and then infected with ~1 MOI of MHV for 24 h, followed by the qRT-PCR analysis of MHV nsp9 mRNA expression. The blots, images, and graphs represent data from at least three independent experiments. The difference between the two groups was calculated using the ANOVA test. Differences were considered statistically significant when P<0.05, *** P<0.001. The blots and graphs represent data from at least three independent experiments.
